# Antioxidant and anti-nociceptive effects of *Phyllanthus amarus* on improving exercise recovery in sedentary men: a randomized crossover (double-blind) design

**DOI:** 10.1186/1550-2783-11-9

**Published:** 2014-03-17

**Authors:** Thapanee Roengrit, Panakaporn Wannanon, Piyapong Prasertsri, Yupaporn Kanpetta, Bung-orn Sripanidkulchai, Naruemon Leelayuwat

**Affiliations:** 1 Department of Physiology, Faculty of Medicine, Khon Kaen University, Khon Kaen 40002, Thailand; 2 Faculty of Allied Health Sciences, Burapha University, Chonburi 20130, Thailand; 3 Exercise and Sport Sciences Program, Graduate School, Khon Kaen University, Khon Kaen 40002, Thailand; 4 Faculty of Pharmaceutical Sciences, Khon Kaen University, Khon Kaen 40002, Thailand; 5 The Exercise and Sport Sciences Research and Development Group, Khon Kaen University, Khon Kaen 40002, Thailand; 6 The Center for Research and Development of Herbal Health Products, Khon Kaen University, Khon Kaen 40002, Thailand

**Keywords:** Herbal plant, Pain threshold, Malondialdehyde, Vitamin C, Muscle damage, Leukocytes, Inflammation

## Abstract

**Background:**

*Phyllanthus amarus* (PA) is a herbal plant containing antioxidant compounds that scavenge free radicals. The reduced oxidative stress may decrease muscle damage leading to early recovery from muscle soreness. This study aimed to evaluate the effects of PA powder on oxidative stress, muscle damage, leukocyte counts, inflammation, and muscle soreness after a single bout of high-intensity exercise.

**Methods:**

Twelve men participated in two 3-day phases separated by a 1-week washout in a randomized double-blinded, crossover design. On day 1, randomly divided participants ingested two capsules of either PA (PA group) or placebo (PLA group) 20 min before a single bout of cycling at high intensity for 20 min followed by four capsules (two capsules after lunch and dinner), and six capsules/day for the next 2 days. Blood samples were collected before, immediately after, and 24 and 48 h after the exercise. Pain threshold was measured at the mid-thigh on both legs.

**Results:**

Malondialdehyde concentration in the PA group was lower than that in the PLA group (p < 0.05) 48 h after high-intensity exercise. Vitamin C concentration was greater in the PA than in the PLA group (p < 0.05) immediately after high-intensity exercise. Pain threshold in both legs in the PA group was higher than in the PLA group 24 and 48 h after high-intensity exercise. There were no significant differences in creatine kinase, leukocyte counts or inflammation between groups.

**Conclusion:**

Acute PA supplementation reduced oxidative stress and muscle soreness induced by high-intensity exercise.

## Introduction

Physical inactivity is the fourth-greatest risk factor for global mortality [1] (World Health Organization [WHO]), because it is an important cause of noncommunicable diseases (NCDs) [2]. To prevent NCDs, WHO introduced the *Global Recommendations on Physical Activity for Health *[3], which encourage people of all ages to begin moderately and gradually progress to higher levels of physical activity. Athletes often perform exercise at high intensity, aiming to win a competition. At the start of an exercise program, muscle damage both in sedentary participants [4] and athletes has often occurred [5]. Exercise-induced muscle damage is attributed to an exercise intensity-dependent increase in oxidative stress [6] produced by mitochondrial process (6). The oxidant damages the plasma membrane, which leads to leakage of creatine kinase (CK) into the blood [7]. This damage generates the inflammatory C-reactive protein (CRP), which facilitates the influx of inflammatory cells to repair the damage [8]. Neutrophils and monocytes then repair the muscle via the oxidative or proteolytic modification to remove tissue debris in the injured muscle [9]. This process, known as the acute inflammatory response, causes muscle soreness after exercise [8]. Alternatively, the muscle soreness may be induced by increased hydrogen ion concentration or decreased pH of the muscle [10]. Rapid recovery from the prior exercise is important for both beginners performing regular exercise to improve their health and athletes preparing for competition. Thus, antioxidant supplementation to attenuate exercise-induced muscle injury, inflammation, and pain may facilitate success in both health promotion and sport competition.

*Phyllanthus amarus* (PA) is a herbal plant widely spread throughout tropical and subtropical areas, including in Thailand. PA extracts contain alkaloids, lignins, flavonoids, and polyphenol compounds [11]. These substances are known to have anti-carcinogenic and anti-inflammatory properties [12], anti-nociceptive effects [13], and hematological and immunological properties [14].

Although eccentric exercise was shown to damage active muscle, concentric exercise was also reported to cause muscular damage [6]. The previous study demonstrated that the high intensity concentric exercise (75% maximal oxygen consumption) increased CK activity immediately after the exercise. Moreover, the high-intensity exercise also increased neutrophil counts 24 h and monocyte counts 2 h after the exercise. These findings showed that the concentric exercise at high intensity causes the damage of active muscles and induces inflammation. Importantly, the reason for applying concentric exercise in this study is that we wanted to imitate real situation in daily life.

Thus far, no research investigating these effects of PA in exercise conditions in humans has been done; thus, we are interested in investigating its antioxidant, anti-inflammatory, and anti-nociceptive effects on improving exercise recovery. To test the hypothesis that acute PA supplementation reduces pain during recovery from high-intensity exercise, we examined the acute effects of PA on oxidative stress, muscle damage, leukocyte counts, inflammation, and muscle soreness after high-intensity exercise.

## Materials and methods

### Participants

Twelve sedentary men aged 22 ± 2.90 years participated in the study. They did not perform regular vigorous exercise and had never taken antioxidant supplements or medications. Prior to enrollment in the study, each subject’s physical examination and electrocardiogram (ECG) were taken. Blood samples were collected after a 12-h overnight fast to measure glucose, creatinine, lipid profiles, and alanine aminotransferase (ALT), to check subjects’ health status prior to the study. None of the subjects was a smoker or had cardiovascular, renal, neuromuscular, orthopedic, or liver disease. This study was approved by the Khon Kaen University Ethical Committee and conformed to the standards set by the Declaration of Helsinki in 2010 (HE531029). All participants provided informed consent to participate in the study after receiving both verbal and written explanations.

### Power calculation

The sample size of this study was calculated by the WINPEPI program by using the study of Fenercioglu et al. [15] which reported that the antioxidant supplement prevent the increased MDA after the exercise. It was decided to require 80% power at a significance level of 0.05. Thus, the proposed size was 12 subjects per group.

### Study design

This study was of a randomized crossover (double-blind) design. Subjects were blinded as to the composition of the PA and PLA or which supplement they were on at which times.

### Supplement preparation

The supplements consisted of PA and placebo (PLA). The aerial parts of PA were collected from Khon Kaen Province. After washing with distilled water and drying at 50°C, the plant was ground and tested for microbial contamination. The plant powder was then placed into capsules containing excipients. One PA capsule contained 100 mg of dried PA powder and one placebo capsule contained Avicel® PH101 (microcrystalline cellulose; FMC BioPolymer, Philadelphia, PA, USA), Aerosil® (desiccant; Evonik Industries, China), and artificial colors. Both supplements were manufactured and controlled the contents by the Center for Research and Development of Herbal Health Products, Khon Kaen University. They were produced in the same lot of manufacture. The dosage of the PA was based on recommendations for the product in Thailand (Khaolaor Laboratories Co. Ltd, G 357/42). The PA contents in each capsule consisted of total polyphenol (33 mg/g) and vitamin C (1.60 mg/100 g) as measured by the Central Laboratory (Thailand) Co. Ltd., Thailand.

### Baseline measurements

Before the experiment, anthropometric and body composition assessments were performed. Body composition was directly measured by Dual emission X-ray absorptiometry (DEXA) in the supine position. Fat distribution was indirectly measured by the ratio of waist and hip circumferences. A 3-day dietary and physical activity records were completed.

### Peak oxygen consumption test

Each participant underwent an incremental exercise test on the cycle ergometer (Corival, Lode, The Netherlands). Oxygen consumption and carbon dioxide production were measured throughout the test using a gas analysis system (AD instrument, ML206, Australia). The test was started with a 12 min 4-stage incremental test in which workload was increased every 3 min for 4 workloads [16]. The workload was determined from an estimate of the participants’ physical fitness status. The peak oxygen uptake value (V̇O_2,peak_) of the participant was determined when any of the following was achieved; the participant’s V̇O_2_ reached a plateau with an increased workload, or a respiratory gas exchange ratio exceeded 1.15, or the heart rate (HR) reached a maximum HR (calculated by the equation: 220 – age), or maximal symptoms of dyspnea and fatigue by using the rating of perceived dyspnea (RPD) and the rating of perceived exertion (RPE) scales, or were unable to maintain cycling at a speed of 60 rpm. HR and ECG were continuously monitored during the test. After the participants reached the criteria, they rested, drank water, and measured their HR until it returned to normal. This test was done at least 1 wk before the initial exercise session.

### Procedure

Participants participated randomly in two 3-day visits 2 weeks apart to prevent carryover effect. At the start of all visits they randomly ingested two capsules of either PA or PLA 20 min before performing exercise on the cycle ergometer. They then exercised without a workload for a 3-min warm-up. They then exercised for 20 min at high intensity (85% of peak oxygen consumption). On the same day and for the next 2 days, participants received the same supplement they had taken initially (two capsules three times per day after meals). Blood samples were collected immediately before ingestion of the supplement, immediately before exercise, and 24 and 48 h after exercise.

### Blood chemistry

At each visit, a 12-mL blood sample was taken from an antecubital vein. After placing 6 mL into EDTA, 2 mL into lithium heparin, and 4 mL into clotting tubes, all samples were placed immediately on ice. One EDTA tube (2 mL) was then added to 1 mol/L of HClO_4_ to precipitate the protein to measure vitamin C. All tubes were centrifuged at 4°C and 3,000 g for 15 min. The upper layer was transferred to a microcentrifuge tube and stored at –80°C until assay. CK, high-sensitivity CRP (hs-CRP), and complete blood count were analyzed in the clinical laboratory of the Srinagarind Hospital, Faculty of Medicine, Khon Kaen University, Thailand.

### Malondialdehyde (MDA) assay

Plasma MDA, a marker of lipid peroxidation, was measured using the thiobarbituric acid (TBA) test according to the method of Draper [17]. The basis of the TBA method is the reaction of MDA with 0.6% of TBA at low pH and 95°C (boiled for 30 min) to form a colored complex. Acid hydrolysis and heat are necessary for the release of MDA bound to the amino groups of proteins and other amino compounds. The MDA-TBA complex, with an absorption at 532 nm, was measured using a spectrophotometer (Genesys 20, SN:35 gk 130009; Thermo Fisher Scientific, Waltham, MA, USA).

### Vitamin C assay

Plasma vitamin C concentration was measured by using Zhang’s method [18]. In this assay, Fe(III) is deoxidized to Fe(II) by ascorbate at pH 4.0 and Fe(II) reacts with potassium ferricyanide to form a blue product, soluble Prussian blue (KFe^III^[Fe^II^(CN)_6_]). The absorbance of this product was monitored over time using a spectrophotometer at 735 nm and the amount of ascorbate was calculated based on absorbance.

### Nitric oxide (NO˙) assay

Plasma NO˙ concentration was measured indirectly using inNO Nitric Oxide Measuring System and Sensors (Innovative Instruments, Inc., Brooks Court, FL, USA). The generation of NO˙ is achieved by the addition of standard nitrite solution to an acidified solution in the presence of a reducing agent such as iodide ion according to the following equation:

$$2\;N{O_{2}}^{‒}+2I^{‒}+4H^{+}\to 2\;\mathrm{NO}˙+I_{2}+2H_{2}O$$

As shown in the chemical equation, the molar ratio of nitrite to nitric oxide is 1:1. Thus, the amount of nitric oxide generated equals the amount of nitrite added.

### Muscle damage

To determine the degree of muscle damage, serum CK activity was assessed. It is proportional to the rate of NADPH formation and was measured with a Roche/Hitachi cobas®c 502 analyzer (Roche, Basel, Switzerland) using standard automated laboratory methods.

### Inflammation

CK activity and hsCRP concentration were determined using a Roche/Hitachi cobas c system (Cobas c 501, Roche Diagnostics, Indianapolis, IN, USA). The principle of CK activity measurement was the rate of the NADPH formation, which is directly proportional to the catalytic CK activity. It is determined by measuring the increase in absorbance. Equimolar quantities of NADPH and ATP are formed at the same rate. The photometrically measured rate of formation of NADPH is directly proportional to the CK activity. HsCRP level was determined using a particle enhanced immuno-turbidimetric assay method, in that agglutinates formation of human CRP with latex particles coated with monoclonal anti-CRP antibody was determined turbidimetrically.

### Muscle soreness

Muscle soreness was measured by the pressure pain threshold of the quadriceps muscle at the mid-thigh using an algometer. Participants were asked to indicate verbally when they felt pain from the pressure.

### Statistics

Data are presented as mean ± SD except when stated otherwise. Dependent variables were analyzed using repeated measure analysis of variance (ANOVA). Statistical analysis was performed using SPSS statistical software, version 18 (SPSS, Chicago, IL, USA). For all statistical tests, differences were considered significant when the p value was less than 0.05.

## Results

### Participant characteristics

The participants in this study were 12 healthy sedentary men. Anthropometry and physical fitness are summarized in Table 1. Baseline blood parameters are shown in Table 2. Regarding percentage of peak oxygen consumption, all participants performed exercise at high intensity (80.62 ± 3.3% and 82.39 ± 7.0 %V̇O_2,peak_ for PLA and PA groups, respectively). In addition, participants’ dietary composition, energy intake, and energy expenditure are presented in Table 3. Physiological parameters, dietary composition (including vitamin C), energy intake, and energy expenditure were not different between groups.

**Table 1:** Anthropometry, body composition and physical fitness of participants

	**Mean ± SD**
Age (yr)	22 ± 2.9
Height (m)	1.69 ± 0.06
BM (kg)	63.6 ± 10.7
BMI (kg/m^2^)	22.8 ± 3.3
%BF	19.5 ± 9.0
FM (kg)	12.5 ± 7.3
FFM (kg)	48.4 ± 6.6
Waist circumference (cm)	76.7 ± 7.5
Hip circumference (cm)	94.8 ± 6.1
Waist: hip circumference ratio	0.81 ± 0.04
**Physical fitness**	
V̇O_2,peak_ (ml/kgBM/min)	36.9 ± 5.2
Work load_max_ (watts)	136.0 ± 32.4

Values are presented as mean ± SD; n = 12.

BM, body mass; BMI, body mass index; %BF, percentage of body fat; FM, fat mass; FFM, fat free mass; V̇O_2,peak_, peak oxygen consumption.

**Table 2:** Baseline blood parameters of participants

	**Mean ± SD**
Blood glucose (mM)	4.6 ± 0.3
Creatinine (μmol/L)	0.01 ± 0.001
ALT (IU/L)	23.08 ± 24.91
TG (mM)	1.1 ± 0.3
TC (mM)	2.6 ± 1.4
LDL (mM)	2.8 ± 0.9
HDL (mM)	1.6 ± 0.4

Values are presented as mean ± SD; n = 12.

TG, triglycerides; TC, total cholesterol; LDL, low density lipoprotein; HDL, high density lipoprotein; ALT, alanine aminotransferase.

**Table 3:** Dietary composition, energy intake and energy expenditure of participants

	**PLA**	**PA**
Dietary composition (/day)		
- Protein (g)	122.9 ± 30.6	125.4 ± 35.6
- Fat (g)	89.3 ± 30.2	78.0 ± 17.4
- Carbohydrate (g)	254.3 ± 71.7	239.2 ± 62.9
- Fiber (g)	5.6 ± 2.0	7.1 ± 2.1
- Vitamin C (mg)	23.2 ± 18.4	27.1 ± 12.2
- Vitamin A (RE)	746.1 ± 1388.8	710.4 ± 1382.4
- Thiamine (mg)	1.3 ± 0.4	1.2 ± 0.5
- Riboflavin (mg)	1.9 ± 1.0	1.4 ± 0.5
- Niacin (mg)	20.7 ± 6.6	18.6 ± 6.5
Energy intake (kcal/day)	2289.2 ± 475.8	2112.0 ± 423.2
Energy expenditure (kcal/day)	2292.1 ± 388.1	2127.9 ± 654.6

Values are presented as mean ± SD; n = 12.

Vitamin A 1 RE = 12 μg alpha-carotene.

PLA, placebo group; PA, *Phyllanthus amarus* group; RE, retinol equivalent; kcal, kilocalories; g, grams; mg, milligrams; kg, kilograms.

### Muscle soreness

**Figure 1:**
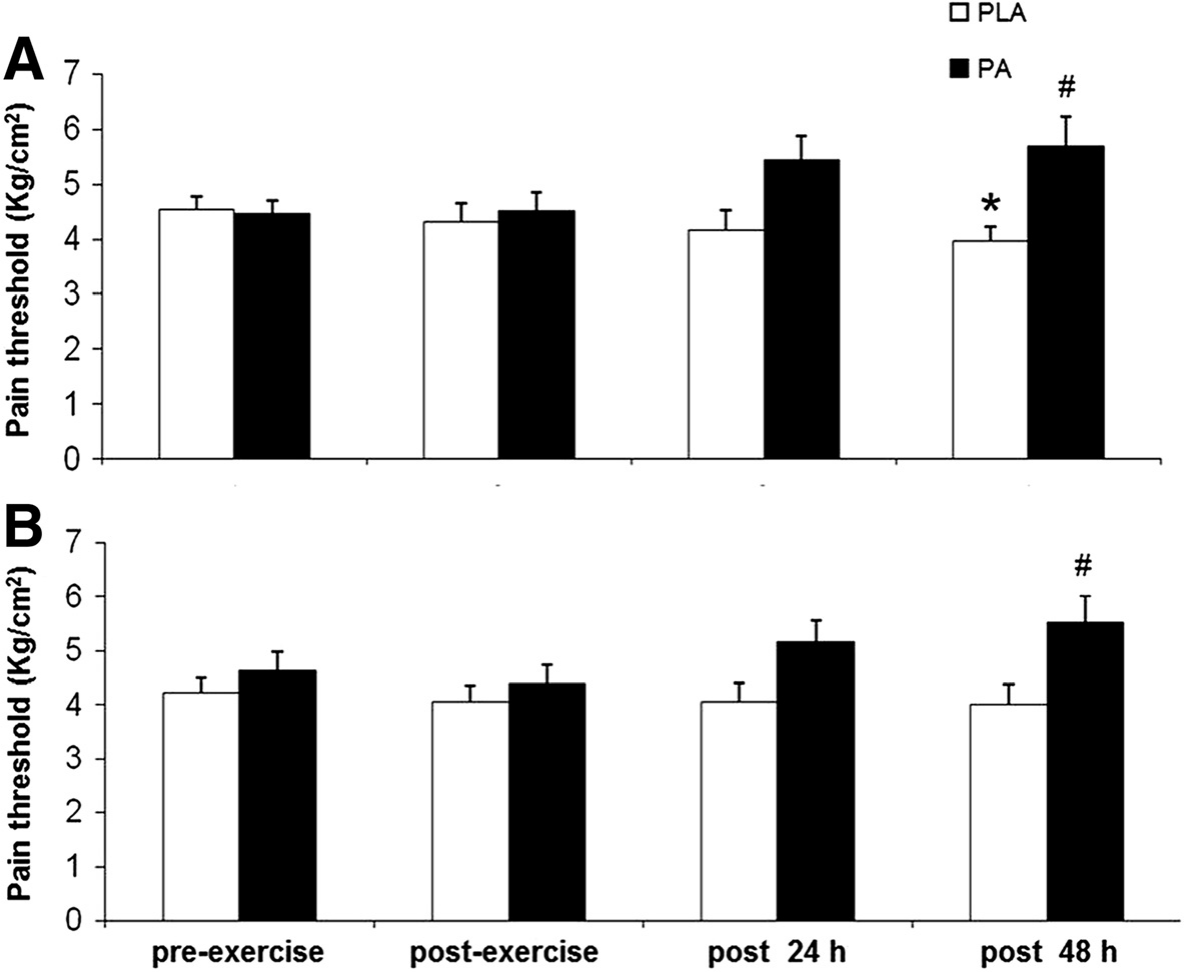
**Levels of pain threshold on the right leg (A) and left leg (B) before exercise (pre-exercise), immediately after (post-exercise), and 24 h (post 24 h) and 48 h (post 48 h) after high-intensity exercise.** Values are presented as mean ± SE (n = 12). PLA, placebo. *Significantly different from before exercise (p < 0.05), #significantly different from the PLA group at the same time point (p < 0.05).

The pain thresholds of both legs were lower at 24 h and 48 h (p < 0.05) after the high-intensity exercise in the PLA group than in the PA group (Figure 1A and 1B). In addition, the pain threshold of the right leg was lower 48 h after high-intensity exercise than pre-exercise in the PLA group (Figure 1A).

### Oxidative stress, damage and inflammation

**Figure 2:**
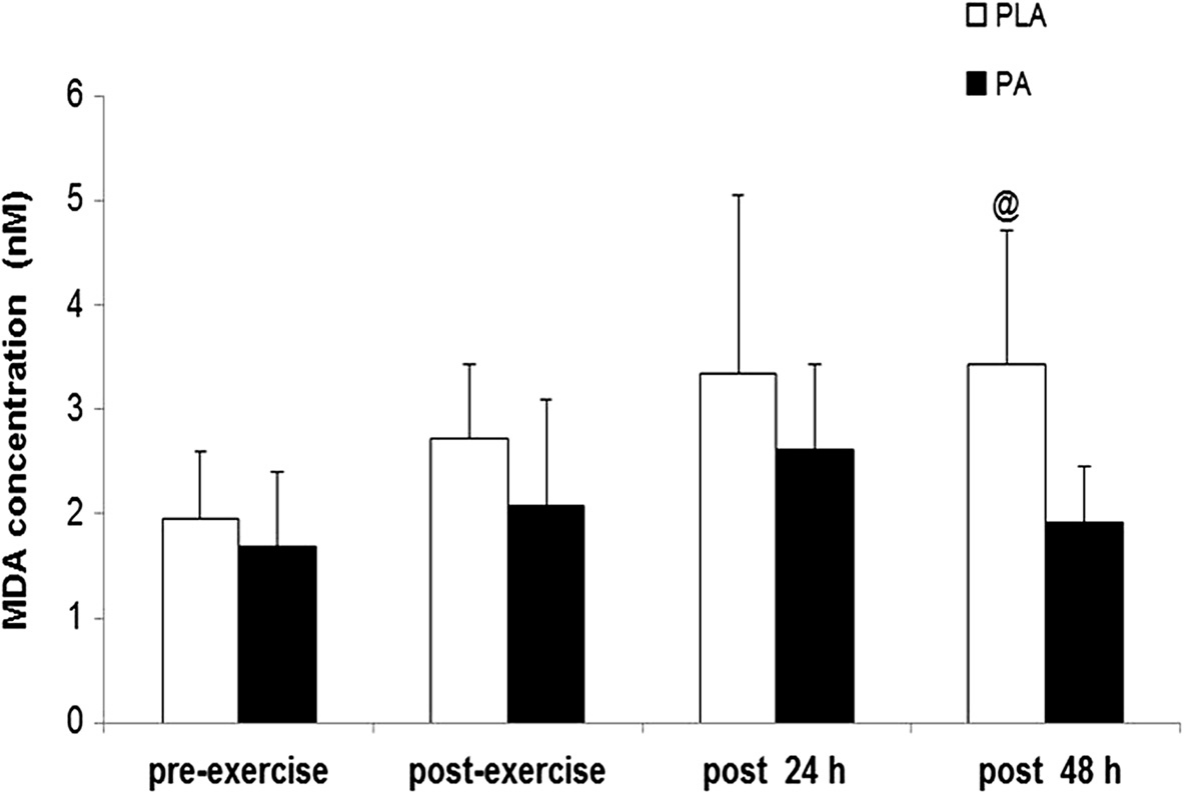
**MDA concentration before exercise (pre-exercise), immediately after (post-exercise), and 24 h (post 24 h) and 48 h (post 48 h) after high-intensity exercise.** Values are presented as mean ± SE (n = 12). MDA, malondialdehyde; PLA, placebo ^@^Significantly different from the PLA group at the same time point (p < 0.01).

**Figure 3:**
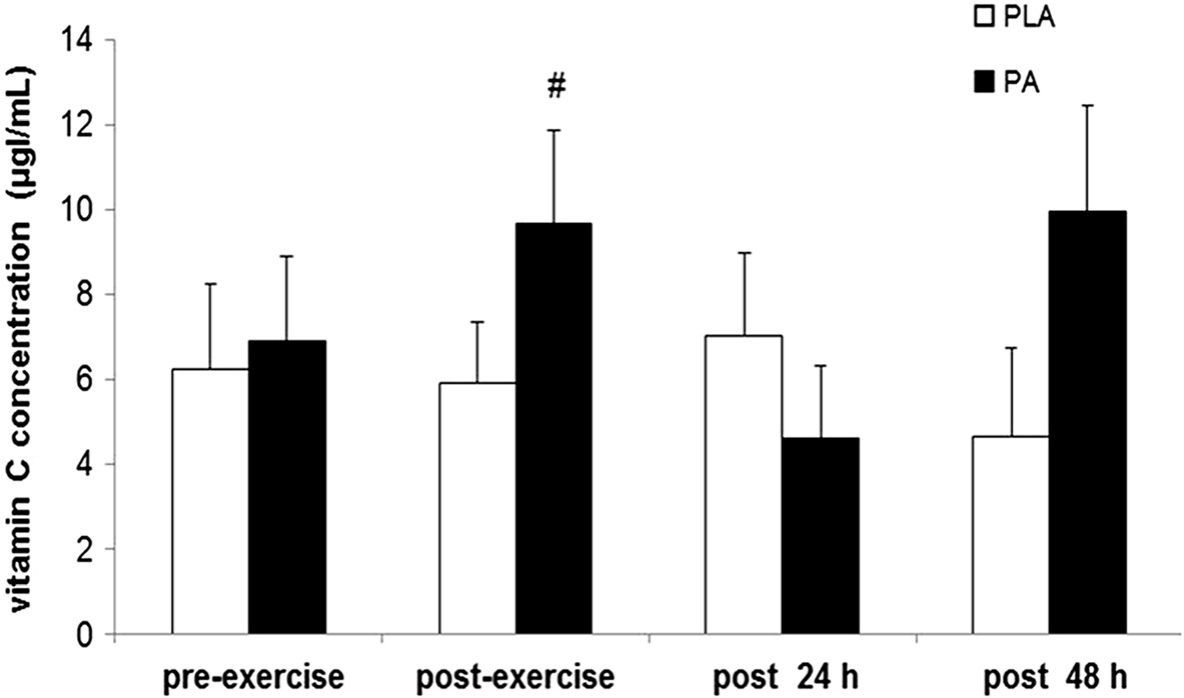
**Vitamin C concentration before exercise (pre-exercise), immediately after (post-exercise), and 24 h (post 24 h) and 48 h (post 48 h) after high-intensity exercise.** Values are presented as mean ± SE (n = 12). PLA, placebo. #Significantly different from the PLA group at the same time point (p < 0.05).

Plasma MDA concentration in the PA group was significantly lower than that of the PLA group 48 h after high-intensity exercise (1.92 ± 0.53 vs. 3.44 ± 1.27 nM; p < 0.01) (Figure 2). Vitamin C concentration was significantly higher in the PA group than in the PLA group immediately after high-intensity exercise (9.68 ± 2.21 vs. 5.91 ± 1.45 μg/mL; p < 0.05) (Figure 3). It then fell to baseline at 24 h after exercise. Plasma CK concentration were significantly higher immediately after exercise than before exercise (Table 4). However, CK, NO˙ and hs-CRP concentrations were not significantly different between groups at any time point (Table 4).

**Table 4:** CK, NO˙, hs-CRP concentrations and leukocyte counts before the exercise (pre-exercise), immediately (post-exercise), 24 h (post 24 h) and 48 h (post 48 h) after the high–intensity exercise

		**Pre-exercise**	**Post-exercise**	**Post-24 h**	**Post-48 h**
CK (U/L)	PLA	151.8 ± 22.4	166.0 ± 24.9*	164.8 ± 25.2	163.7 ± 30.1
	PA	132.3 ± 19.0	194.5 ± 21.5*	182.6 ± 41.5	145.6 ± 65.6
hs-CRP (mg/L)	PLA	1.24 ± 0.37	1.36 ± 0.43	1.58 ± 0.74	2.28 ± 1.11
	PA	1.24 ± 0.33	1.15 ± 0.33	1.57 ± 0.57	1.26 ± 0.42
Neutrophils (10^3^/μL)	PLA	3.68 ± 0.33	4.80 ± 0.61*	4.05 ± 0.53	3.66 ± 0.19
	PA	3.53 ± 0.35	4.39 ± 0.45*	3.09 ± 0.25∞	3.37 ± 0.26
Lymphocytes (10^3^/μL)	PLA	2.08 ± 0.19	2.57 ± 0.39	1.77 ± 0.08∞	1.75 ± 0.10
	PA	1.88 ± 0.16	2.61 ± 0.28*	2.13 ± 0.15	1.94 ± 0.09∞
Monocytes (10^3^/μL)	PLA	0.41 ± 0.02	0.45 ± 0.05	0.44 ± 0.06	0.43 ± 0.03
	PA	0.31 ± 0.44	0.36 ± 0.41	0.48 ± 0.33∞	0.44 ± 0.06

Values are presented as mean ± SE; n = 12.

NO˙, nitric oxide; hs-CRP, high sensitive C-reactive protein.

*Significantly different from before the high–intensity exercise (p < 0.05).

∞Significantly different from immediately after the exercise (post-exercise) (p < 0.05).

### Leukocyte counts

Neutrophil count was significantly higher immediately after exercise than before exercise in both groups (Table 4). Neutrophil count at 24 h was significantly lower than immediately after exercise in the PA group (p < 0.05) (Table 4). Lymphocyte count significantly increased immediately after exercise when compared with pre-exercise (p < 0.05) (Table 4). Moreover lymphocyte counts at 48 h in the PA group and 24 h in the PLA group significantly lower than immediately after the exercise (p < 0.05) (Table 4). Monocyte count was significantly increased 24 h after the exercise when compared with pre-exercise (p < 0.05) (Table 4). There were no significant between-group differences in leukocyte count at any time point.

## Discussion

Our results demonstrate that acute PA supplementation may reduce the muscle soreness of both legs during recovery from high-intensity exercise in healthy participants.

The results partially support our hypothesis that acute PA supplementation reduces pain during recovery from high-intensity exercise. Previous studies on PA supplementation have demonstrated the antioxidant and anti-nociceptive effects of PA in animals under non-exercise conditions [19-21]. Thus, the present study is the first to demonstrate a reduction in muscle pain after the ingestion of PA immediately before and a few days after the exercise with improved oxidative stress during recovery. A review of the literature suggests that muscle soreness is strongest 24–72 h after exercise [22]. Our finding of decreased muscle soreness at 24 h may demonstrate a potential effect of PA on recovery from muscle soreness and may enable beginners in sports activity to return to a competition or an exercise session the next day.

PA has been shown by this study to contain a wide variety of antioxidant compounds, such as vitamin C and total polyphenol compounds. The lack of difference in dietary vitamin C intake before the experiment between the groups confirmed that the greater plasma vitamin C concentration was induced by the PA supplementation. The amount of vitamin C in the PA intake per day in this study seemed to be too small to increase plasma vitamin C concentration. In a previous study, vitamin C intake of less than 200 mg (9.60 mg in this study) increased the absorption rate to 98% [23]. This may contribute to the higher vitamin C concentration immediately after the exercise in the PA than the placebo group. Apart from vitamin C, the PA in each capsule contains 33 mg/g of total polyphenol. Although polyphenol compounds in participants’ blood were not measured in this study, it may play antioxidant role during and immediately after the exercise. Polyphenol may spare the antioxidant activity of vitamin C at this period. Then the decreased vitamin C concentration at the following 24 h after the exercise may imply that vitamin C was greatly consumed in scavenging free radicals resulting in the decrease of lipid peroxidation indicated by MDA at 48 h after the exercise.

In addition, three possible mechanisms explaining important anti-nociceptive role of vitamin C and total polyphenol compounds induced by PA supplementation are: 1) stimulation of the secretion of beta-endorphin by vitamin C, which is effective at reducing neuropathic pain [24], 2) scavenging reactive oxygen species [25], which play an important role in reducing neuropathic pain [26]. The reduction in free radicals resulted in decreased prostaglandins [27], which are sympathetic nervous system pain mediators [28], as well as by central inhibitory pain mechanisms [29]. Unfortunately, beta-endorphin secretion and the pain mediators are not examined by this study. Therefore, the effects of acute PA supplementation on either beta-endorphin secretion or the liberation of pain mediators should be further investigated. 3) Reduction in hydrogen ion produced by high-intensity exercise may be one of the mechanisms that explain the effect of these antioxidants on pain reduction. Proton normally produced by high-intensity exercise was shown to increase pain and fatigue [30]. Recently polyphenol including flavonoid was demonstrated to scavenge free radicals by hydrogen ion transfer. This may thus reduce noxious effects due to oxidative stress [31].

The anti-nociceptive effect of antioxidants mentioned above was shown in non-exercise condition. This effect on exercise recovery in humans is still unclear. Some authors have reported reductions in muscle pain [32], but others did not demonstrate this effect [33,34]. The discrepancy may be due to the duration and timing of supplementation. The anti-nociceptive effect seems to be present if PA is ingested long before the exercise (>2 wk) [32,35,36] because this finding is not reported in previous studies with shorter durations of pre-exercise (2 h to 3 d) and post-exercise supplementation [33,34,37-39].

The significant increase in plasma CK concentration indicated increased muscle damage, as determined by both supplement groups immediately after the high-intensity exercise. This indicates that the single bout of high-intensity exercise for 20 min in this study was sufficient to cause muscle damage. However, we did not find any difference in CK concentration between groups, which, combined with the lack of differences in immune cell counts and inflammatory responses to the exercise between groups suggests that the development of cell damage and inflammation in this exercise was not attenuated by PA supplementation.

Improved oxidative stress after exercise by PA supplementation might be useful for some medical conditions generating high oxidative stress, such as type 2 diabetes mellitus [40] or for individuals with pre-hypertension who got benefit effect from the reduced oxidative stress [41] but further study would be required to prove this possibility. It is likely that acute supplementation during exercise may provide protection until patients can adapt to oxidative stress by modifying their own antioxidant status, which require a bit of time. Moreover, prolonged supplementation before exercise may provide protective effects against oxidative stress and exercise recovery in the patients who are not trained person.

We performed our research according to the guidelines proposed by a recent review article on studies investigating the impact of antioxidant supplementation on exercise performance [42] by carefully preparing and storing the antioxidant compounds; i.e., PA in this study, to prevent oxidation and degradation. Also, to ensure that the results in this study were really due to the PA ingested, we compared the dietary status of both groups and confirmed no difference between them. Moreover, the subjects had no other antioxidant supplementation at any time before or during the experiment.

A limitation of this study is that the dose of PA powder was the dose for resting conditions. This was intended to avoid adverse effects on participants. A longer duration of PA supplementation before exercise, or a higher dose of PA, might be required to yield muscle damage and inflammation and greater and earlier beneficial effects on muscle soreness after high-intensity exercise, which in turn may contribute to rapid recovery after high-intensity exercise.

## Conclusions

The present study shows that PA supplementation improved oxidative stress and muscle soreness induced by high-intensity exercise. This may encourage beginners or novices in sports activity or fitness to return to a competition or an exercise session the next day, with early recovery from muscle soreness. The present results add a treatment value of PA, a herb generally found in Thailand, in aspects of both health and sport.

## Abbreviations

ALT: Alanine aminotransferase; ANOVA: Analysis of variance; CK: Creatine kinase; ECG: Electrocardiogram; DEXA: Dual emission x-ray absorptiometry; EDTA: Ethylene diamine tetra acetic acid; HDL: High-density lipoprotein; hs CRP: high sensitive C reactive protein; MDA: Malondialdehyde; NO˙: Nitric oxide; NCDs: noncommunicable diseases; PA: *Phyllanthus amarus*; PLA: Placebo; RE: retinol equivalent; TBA: Thiobarbituric acid; V̇O2,peak: Peak oxygen consumption; WHO: World Health Organization.

## Competing interests

The authors declare that they have no competing of interest.

## Authors’ contributions

NL conceived and designed the study and prepared the manuscript. PW provided medical coverage throughout the experiment. TR carried out all the experimental work and statistical analysis and helped to draft the manuscript. PP and YK assisted the experimental work. BS provided PA and PLA supplements. All authors read and approved the final manuscript.
